# Biomarkers for Hepatocellular Carcinoma: From Origin to Clinical Diagnosis

**DOI:** 10.3390/biomedicines11071852

**Published:** 2023-06-28

**Authors:** Mona A. Omar, Mohamed M. Omran, Khaled Farid, Ashraf A. Tabll, Yasser E. Shahein, Tarek M. Emran, Ana Petrovic, Nikola R. Lucic, Robert Smolic, Tanja Kovac, Martina Smolic

**Affiliations:** 1 Chemistry Department, Faculty of Science, Damietta University, New Damietta 34517, Egypt; monaomar9050@yahoo.com; 2 Chemistry Department, Faculty of Science, Helwan University, Cairo 11795, Egypt; drmmomran@science.helwan.edu.eg; 3 Tropical Medicine Department, Faculty of Medicine, Mansoura University, Mansoura 35524, Egypt; khaledfarid68@yahoo.com; 4 Microbial Biotechnology Department, National Research Centre, Cairo 12622, Egypt; 5 Immunology Department, Egypt Center for Research and Regenerative Medicine (ECRRM), Cairo 11517, Egypt; 6 Molecular Biology Department, National Research Centre, Cairo 12622, Egypt; 7 Clinical Pathology Department, Faculty of Medicine, Al-Azhar University, New Damietta 34517, Egypt; drtarekemran@yahoo.com; 8 Faculty of Dental Medicine and Health Osijek, Josip Juraj Strossmayer University of Osijek, 31000 Osijek, Croatia; anapetrovic@fdmz.hr (A.P.); nrlucic@fdmz.hr (N.R.L.); rsmolic@fdmz.hr (R.S.); tanjakovac@fdmz.hr (T.K.)

**Keywords:** hepatocellular carcinoma, diagnosis, imaging methods, molecular markers

## Abstract

The incidence of hepatocellular carcinoma (HCC) and HCC-related deaths has increased over the last few decades. There are several risk factors of HCC such as viral hepatitis (B, C), cirrhosis, tobacco and alcohol use, aflatoxin-contaminated food, pesticides, diabetes, obesity, nonalcoholic fatty liver disease (NAFLD), and metabolic and genetic diseases. Diagnosis of HCC is based on different methods such as imaging ultrasonography (US), multiphasic enhanced computed tomography (CT), magnetic resonance imaging (MRI), and several diagnostic biomarkers. In this review, we examine the epidemiology of HCC worldwide and in Egypt as well as risk factors associated with the development of HCC and, finally, provide the updated diagnostic biomarkers for the diagnosis of HCC, particularly in the early stages of HCC. Several biomarkers are considered to diagnose HCC, including downregulated or upregulated protein markers secreted during HCC development, circulating nucleic acids or cells, metabolites, and the promising, recently identified biomarkers based on quantitative proteomics through the isobaric tags for relative and absolute quantitation (iTRAQ). In addition, a diagnostic model used to improve the sensitivity of combined biomarkers for the diagnosis of early HCC is discussed.

## 1. Hepatocellular Carcinoma

### 1.1. Epidemiology of Hepatocellular Carcinoma

Approximately 690,000 cases of hepatocellular carcinoma (HCC) are diagnosed annually [1]. About 80–90% of all HCC cases are closely related to chronic liver diseases (CLD) and cirrhosis of the liver. In the past three decades, it has been shown that viral infections such as hepatitis B (HBV) and C viruses (HCV), alcoholic liver disease, as well as nonalcoholic fatty liver diseases are the major risk factors for liver cirrhosis [2]. A total of 54% and 31% of HCC cases are attributed to HBV and HCV, respectively, while 15% of HCC cases are related to other causes [3].

Age, ethnicity, gender, activity level, regional distribution, and stage of underlying liver disease all affect the global incidence of HCC differently [4,5]. Globally, cases of HCC in individuals under the age of 40 years are uncommon, while the peak occurs around the age of 70. HCC is more frequent in males than females, indicating gender predilection and the difference being much greater in high-risk areas. Two factors account for this disparity. First, men are generally more exposed to hepatitis-virus- and liver-cancer-causing substances, such as alcohol and cigarettes. Second, women’s estrogen hormone may limit interleukin-6-mediated inflammation, reducing hepatic injury and compensatory proliferation, giving the impression that they have a lower risk of developing HCC. Contrarily, testosterone in men has been shown to stimulate androgen receptor signaling, which supports the growth of HCC cells [6].

Geographically, the incidence of HCC varies widely; 30 cases per 100,000 individuals are found in Southeast Asia and Central Africa, and more than 80% of HCC patients are found in Sub-Saharan Africa and East Asia [2,4]. With an annual incidence ranging from 2% to 8%, cirrhosis caused by HCV is the main risk factor for developing HCC in Europe, Japan, Latin America, and the United States. The main risk factor in China is HBV infection [7,8].

### 1.2. Epidemiology of HCC in Egypt

HCC is a significant health issue in Egypt. As the sixth-largest nation in the Middle East and the Arab world, the prevalence of hepatocellular carcinoma has increased from 4% to 7.2% over the past ten years [8]. Patients with chronic liver disorders have had a striking increase in HCC. Compared to other nations on the planet, Egypt has the greatest prevalence of HCV, with 14.7% of the general population being affected. According to estimates [9], 40–50% of HCC cases are associated with HCV [10]. Because HCV infection is so common, anti-schistosomal treatment with tartar emetic injections is being considered [9,11]. However, the main geographical differences in risk factors for chronic liver diseases between Egypt and other countries include a high prevalence of HCV infection, a significant burden of nonviral liver diseases such as fatty liver disease and autoimmune liver diseases, and a relatively high prevalence of metabolic syndrome. Antiviral treatments can have a significant impact on the progression and outcome of chronic liver diseases caused by HBV and HCV. Treatment goals include the sustained suppression of viral replication, a cure for the infection (in the case of HCV), and a reduction in liver-related mortality and morbidity [12,13]. It is worth mentioning that autoimmune liver diseases such as autoimmune hepatitis (AIH) and primary biliary cholangitis (PBC) can increase the risk of HCC development, particularly in the presence of cirrhosis. Regular monitoring for HCC in these patients is important for early detection and treatment [14].

## 2. Diagnosis of HCC

For a correct diagnosis of HCC, serum biomarkers, one or more imaging investigations, and histology and immunostaining confirmation are all necessary [15].

### 2.1. Imaging Methods

#### 2.1.1. Ultrasonography (US)

Ultrasound is the first selection method for HCC because it is low-cost, noninvasive, can be repeated every 3 to 6 months without any risk, and is well accepted by patients. The sensitivity for HCC is 58–70% and it cannot detect HCC tumors less than 1 cm in size [16].

#### 2.1.2. Multiphasic Enhanced Computed Tomography (CT)

A computed tomography (CT) scan is a three-dimensional reconstruction technique and a public test for detecting HCC with higher sensitivity than US (53–87%) for early HCC [17].

#### 2.1.3. Magnetic Resonance Imaging (MRI)

**Figure 1:**
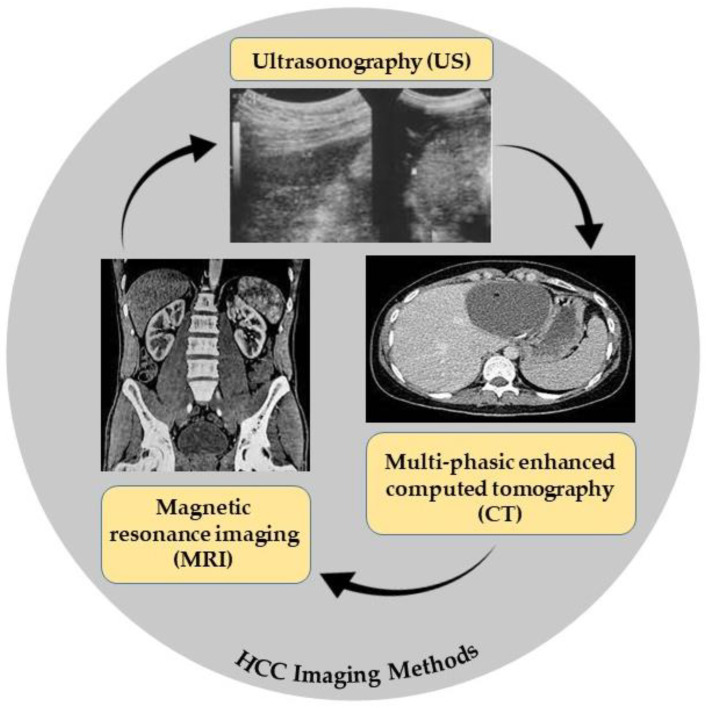
HCC imaging methods.

MRI is superior in both the identification and characterization of HCC, and it is still improving. CT and MRI both have 90% sensitivity for tumors under 2 cm. However, MRI is more sensitive than CT for HCC diagnosis (80–92%), especially for extra nodules between 1 and 2 cm in size (84% vs. 47%). Because of its limited availability and high cost, MRI is only used for characterization, diagnostic confirmation, and intrahepatic tumor staging. When patients have equivocal results with US as their first modality, CT and MRI are preferable [18]. The imaging methods used in HCC diagnostic are demonstrated in Figure 1.

### 2.2. Liver Tissue Biopsy

If HCC manifests in a patient who is not cirrhotic and imaging methods are insufficient to identify HCC, liver biopsy may be required. For tumors larger than 1 cm, the American Association for the Study of Liver Diseases (AASLD) does not advise biopsy in cases when the results of two separate imaging modalities are in agreement. Liver biopsy has a sensitivity of 65–95%, absolute specificity, and PPV. Several complications are possible, such as pain, puncture of the gallbladder, bile peritonitis, and severe bleeding.

The main approach for diagnostic liver tissue assessment involves a comprehensive examination of hepatic tissue stained with hematoxylin and eosin (H&E). Additional special stains may be used to highlight or identify features that are not easily visible with H&E staining. Masson’s trichrome stain is commonly used for staining biopsy-obtained hepatic tissue, and immunostaining with polyclonal and monoclonal antibodies is also an option. However, compared to the histochemical staining methods mentioned above, immunohistochemical approaches are not frequently used in the diagnostic assessment of non-neoplastic liver diseases [19]. The diagnostic capacity of MRI was greatly enhanced to detect HCC, using several classes of contrast agents. These agents include hepatocyte-selective and reticuloendothelial-system-targeted agents such as gadobenate dimeglumine (Gd-BOPTA), mangafodipir trisodium (Mn-DPDP), and the most recently discovered hepatocyte-specific agent, gadoxetic acid (Gd-EOB-DTPA). These agents show significant paramagnetic properties to shorten liver longitudinal relaxation time, leading to enhanced sensitivity in the noninvasive detection of small hepatic lesions [20].

### 2.3. Liquid Biopsy

**Figure 2:**
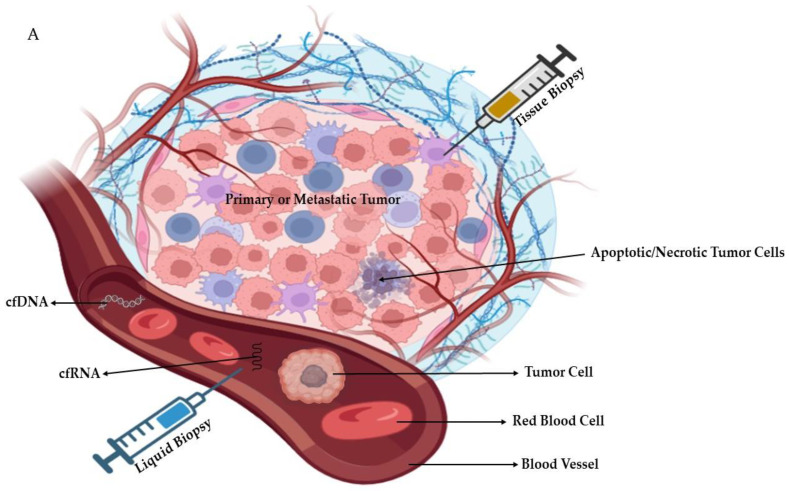

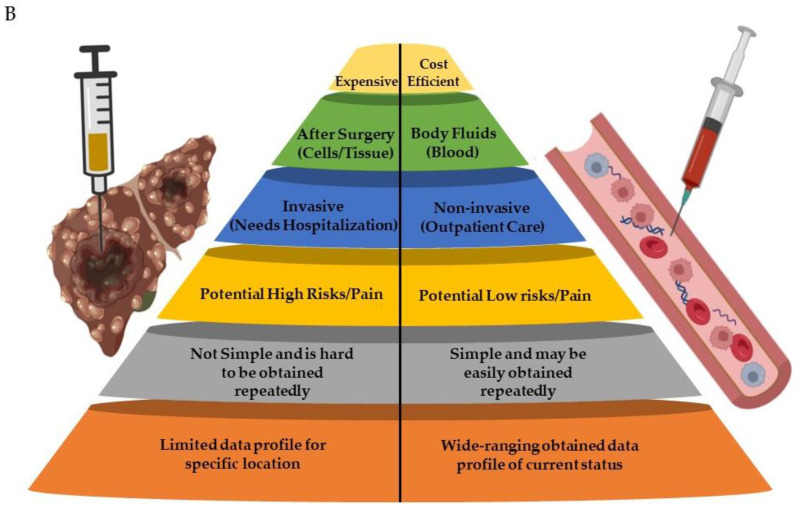
(**A**): Schematic diagram showing tissue and liquid biopsies with the examples of blood-circulating factors (cell free-RNA/-DNA, cells, etc.). (**B**): Comparison between liver tissue and liquid biopsies. Parts of the figure were generated using BioRender.com.

A liquid biopsy is indicated to examine tumor elements that are present in the bloodstream. Potential issues with tissue samples, noninvasiveness, and lower costs are all advantages of liquid biopsy [21]. Repeating liquid biopsies is simple throughout HCC treatment. Circulating tumor cells (CTCs), circulating cell-free DNA integrity, somatic mutations, circulating cell-free tumor DNA methylation, and circulating RNA are all examples of liquid biopsy markers [22], as shown in Figure 2A. A summary of the potential differences between tissue and liquid biopsies is shown in Figure 2B.

### 2.4. Biomarkers

**Figure 3:**
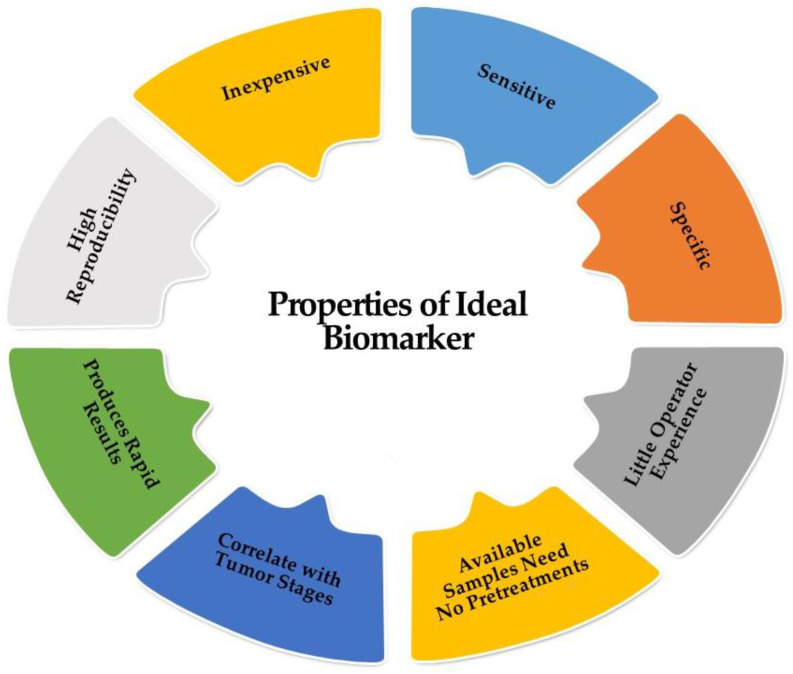
Characters of the ideal biomarker.

**Figure 4:**
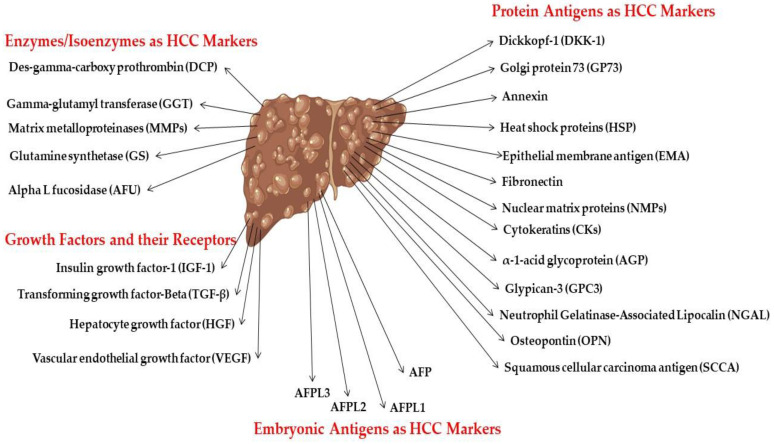
HCC marker classification according to biochemical nature.

A diagnostic biomarker is defined as any substance, structure, or process that can be detected, measured, or quantified in the body and aims to indicate or predict the occurrence of a disease, as well as its progression or treatment efficiency. The ideal biomarker has certain universal properties for routine clinical analysis (sensitive; specific; does not require much operator experience; inexpensive; highly reproducible; produces rapid results; correlates with tumor stages; and available samples (should be readily available like blood or urine) need no pretreatment); these are demonstrated in Figure 3 [23]. HCC marker classification according to the biochemical properties is illustrated in Figure 4.

#### 2.4.1. Embryonic Antigen

Alpha-fetoprotein (AFP) is a 70 kDa glycoprotein. It is highly expressed in the human fetus growth process and its synthesis ends after birth [24]. During liver cancer, AFP synthesis is highly expressed. Metabolites such as bilirubin, steroids, and fatty acids, as well as various drugs, circulate by binding to AFP. For HCC detection, the cutoff levels are more than 400 U/L. The sensitivity of AFP ranges from 20 to 60%, and specificity ranges from 80 to 100%.

Based on their capacity to bind to the lectin Lens culinaris agglutinin (LCA), AFP can be divided into three isoforms: AFP-L1 (nonbinding fraction), AFP-L2 (weak binding fraction), and AFP-L3 (binding fraction). Early in the course of hepatic illness, AFP-L1 levels rise and AFP-L2 has a more moderate affinity for lectin. Because HCC cells are the only ones that can produce AFP-L3, it is only elevated in HCC patients, making it a diagnostic biomarker for HCC. These findings identify hs-AFP-L3 as a valuable biomarker for the early identification of HCC [25].

#### 2.4.2. Protein Antigens

**A.**  **Dickkopf-1 (DKK-1)**

DKK-1 is a 266-amino-acid (35 kDa) secreted glycoprotein that acts as a secretary antagonist of the Wnt signaling pathway. It is a promising diagnostic biomarker for HCC and can be used in combination with AFP for the diagnosis of HCC, particularly in those cases with low AFP concentration [26].

**B.**  **Golgi protein 73 (GP73)**

GP73 is a 73 kDa transmembrane glycoprotein. It is often expressed in healthy biliary epithelial cells and located inside the Golgi complex, but not in healthy hepatocytes [27,28]. Patients with hepatocellular carcinoma and hepatic cirrhosis have increased levels of GP73 expression. For the detection of early HCC in cirrhotic patients, GP73 is superior to AFP [29]. In cases of HCV-related cirrhosis, serum GP73 may be a reliable diagnostic biomarker for the early identification of HCC [30]. In comparison to using each test alone, combining GP73, DKK-1, and AFP increased the sensitivity and specificity for the diagnosis of HCC [31].

**C.**  **Annexin**

This is a phospholipid-binding protein which enhances cell motility, proliferation, invasion, adhesion, and metastasis. An indication of the overall severity of HCC, annexin A2 overexpression is involved in the growth and metastasis of HCC. Annexin A2 has greater sensitivity and specificity than AFP for the identification of HCC [32].

**D.**  **Heat shock proteins (HSP)**

HSPs are stress-induced proteins that are produced in response to general stressors like carcinogenesis. The apoptotic process and the immunological response to tumors have both been linked to HSP expression. HSP70, in particular, has been identified as a viable and sensitive marker to distinguish early HCC from precancerous lesions [33,34].

**E.**  **Epithelial membrane antigen (EMA)**

EMA is a mucintransmembrane glycoprotein found on epithelial surfaces. EMA expression can be used to help diagnose liver cancer [35].

**F.**  **Fibronectin**

Hepatocytes produce fibronectin, which is found in the extracellular matrix. Integrins, collagen, heparin sulphate proteoglycan, and fibrin are all interconnected with fibronectin. This high-molecular-weight glycoprotein mediates cell adhesion, growth, differentiation, and migration. Abnormal fibronectin expression promotes HCC development [35,36].

**G.**  **Nuclear matrix proteins (NMPs)**

NMPs are components of the nucleus’s structural framework and are released by dead cells’ nuclei. NMPs are investigated as a biomarker of organ injury in liver illnesses. This is due to the fact that the nuclear shape is altered along with the neoplastic transformation as a result of changes in the structure or composition of the nuclear matrix proteins [37].

**H.**  **Cytokeratins (CKs)**

CKs comprise a group of intermediate filamentous cytoplasmic cytoskeletal proteins, forming the cytoskeleton of epithelial cells. CKs act in order to maintain normal epithelial cell integrity. Because CKs are the most abundant intermediate filament proteins in the liver, any cellular damage that compromises the integrity of hepatocytes may result in the release of CKs into the bloodstream [38,39].

**I.**  **α-1-acid glycoprotein (AGP)**

AGP is primarily produced by hepatocytes as a glycosylated acute-phase protein with a molecular mass of 41–43 kDa. Cytokines can raise AGP levels as part of an inflammatory response. During HCC and cirrhosis, modifications in the sialylation and fucosylation of AGP may take place, which targets AGP as a blood biomarker [40,41].

**J.**  **Glypican-3 (GPC3)**

GPC3, a 60 kDa heparan sulphate proteoglycan anchored to the cell membrane, is not expressed in adult livers. Growth and development are strongly linked to the GPC3 protein. Compared to individuals with liver cirrhosis caused by HCV infection, GPC3 serum levels are higher in HCC patients. Nevertheless, GPC3 is more sensitive than AFP in detecting small, well-differentiated HCCs. In comparison to using each marker separately, it has been demonstrated that using GPC3 and AFP together provides significant sensitivity and specificity. For the diagnosis and treatment of HCC, glypican-3 is developing into a promising diagnostic biomarker and therapeutic target [42,43].

**K.**  **Neutrophil Gelatinase-Associated Lipocalin (NGAL)**

NGAL, or Lipocalin 2 (LCN2), is a glycoprotein that belongs to the lipocalin subfamily and has a molecular mass of 25 kDa. There are additional forms of NGAL in serum, including a 30 kDa isoform that most likely originates from variable glycosylation, a 46 kDa homodimer linked by a disulfide, and a 130 kDa heterodimer that binds to the proMMP-9, the inactive form of the matrix metalloproteinase-9 (MMP-9) [44]. Tumor invasion and metastasis are significantly influenced by MMP-9. Dertli et al. [45] showed that the NGAL/MMP9-bound form protects MMP9 from degradation and, hence, boosts its activity [45]. NGAL was first identified as a part of MMP-9 in the specialized granules of neutrophils [46]. Later research, however, revealed that lipocalin 2 is also synthesized in other organs, including the kidney under tubular injury and the liver when there is liver cell injury or regeneration. NGAL may, therefore, be a possible biomarker of liver damage. Recently, it was found that NGAL released in urine is a noninvasive diagnostic marker for HCC detection and that patients with chronic liver disease would benefit from using urinary NGAL to diagnose HCC [47].

**L.**  **Osteopontin (OPN)**

OPN is a glycophosphoprotein that binds to integrins. Numerous physiological processes, including immunological and inflammatory reactions, antiapoptosis, control of cell survival, cell invasion and migration, and tumor metastasis, are all functionally regulated by widespread OPN expression. In patients with HCC, OPN is elevated and it was marked to be more specific but less sensitive than AFP in HCC diagnosis [48,49].

**M.**  **Squamous cellular carcinoma antigen (SCCA)**

This is a serine protease inhibitor with a molecular mass of 48 kDa. Typically, SCCA is expressed by neoplastic cells of epithelial origin and is detected in HCC tissues. It protects tumor cells from apoptosis. Clinically, SCCA was useful in identifying HCC patients. SCCA increased AFP’s capacity to diagnose HCC by up to 90% [50,51].

**Figure 5:**
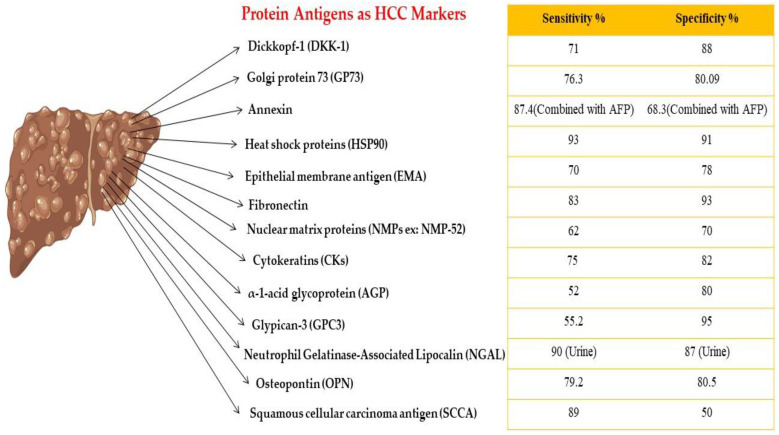
Protein antigens as HCC biomarkers.

The sensitivity and specificity of different potential HCC markers as protein antigens and listed from A to M are shown in Figure 5.

#### 2.4.3. Enzymes and Isoenzymes as HCC Biomarkers

**A.**  **Des-gamma-carboxy prothrombin (DCP)**

DCP is an abnormal prothrombin lacking γ-carboxy residues, which impairs its clotting function. This protein is induced by vitamin K absence or antagonist II (PIVKA II). Malignant hepatocytes lack the ability to carboxylate glutamic acid to create γ-carboxy glutamic acid. Des-γ-carboxy prothrombin or aberrant prothrombin is formed as a result of this failure. Compared to AFP, DCP levels showed greater sensitivity and specificity in distinguishing HCC from chronic nonmalignant hepatic diseases. The sensitivity of HCC detection was increased by combining DCP and AFP [52,53].

**B.**  **Gamma-glutamyl transferase (GGT)**

In healthy individuals, this enzyme is mostly secreted by Kupffer cells, the liver’s resident macrophages, and bile duct endothelial cells. A GGT unique to hepatoma is termed GGT-II. In HCC, there are significant alterations in serum GGT activity. When compared to using AFP alone, the combination of GGT and AFP showed a higher sensitivity compared to lower specificity [54].

**C.**  **Matrix metalloproteinases (MMPs)**

MMPs are calcium-dependent zinc-containing endopeptidase enzymes that regulate the integrity and degrade the components of the extracellular matrix (ECM), involved in proliferation, differentiation, apoptosis, adhesion, migration, wound healing, and tissue regeneration. During the progression of hepatic diseases, MMPs degrade the ECM and induce cytokine release and fibrosis progression, which may be resolved or proceed to form HCC. Most liver cells have high levels of MMP-2, -3, -7, and -16 during viral hepatitis and HCC; MMP-8, -9, -10-, -12, -13, -14, and MMP-16 during HCC and cirrhosis; and MMP-24 during liver regeneration. On the contrary, other MMPs have a low level of expression such as MMP-1 in viral hepatitis and HCC, and MMP-15 during liver regeneration [55]. Selective MMP inhibitors have the ability to halt the spread and growth of HCC [56].

**D.**  **Glutamine synthetase (GS)**

The enzyme GS is crucial for the synthesis of glutamine from glutamate and ammonia as well as for hepatic cells’ nitrogen balance and ammonia metabolism. Increased GS mRNA expression has been linked to metastasis and cancer progression in HCC [57].

**E.**  **Alpha L fucosidase (AFU)**

All mammalian cell lysosomes contain AFU, a glycosidase that hydrolyzes the fucose glycosidic bonds of glycoprotein and glycolipids. It is involved in the degenerative response of a number of fucoglyco-containing complexes [58].

**F.**  **Paraoxonase 1 (PON1)**

**Figure 6:**
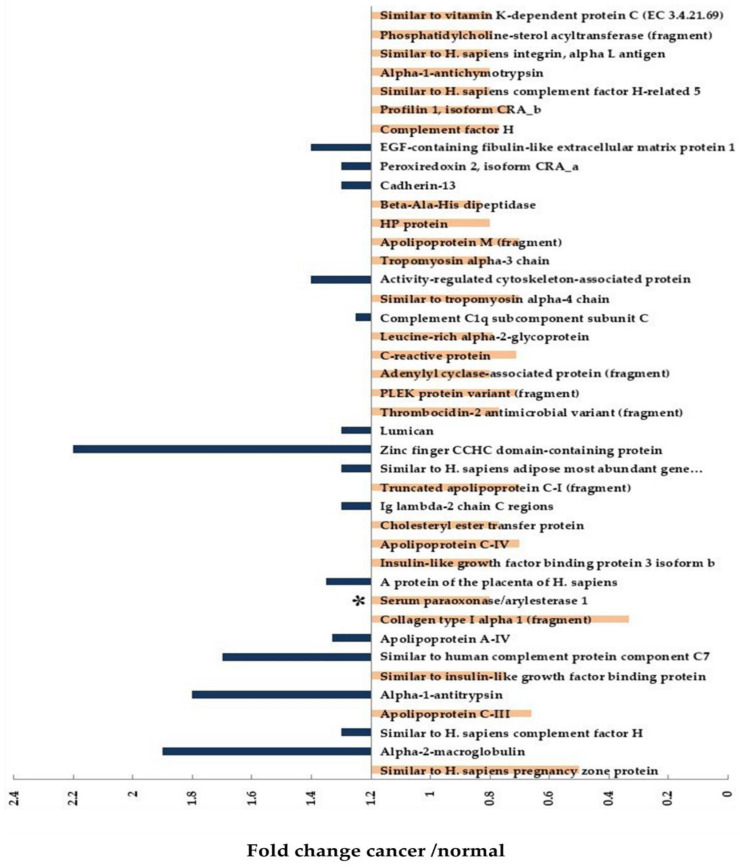
A total of 41 differentially expressed proteins in HCC and identified using quantitative proteomics through the isobaric tags for relative and absolute quantitation-(iTRAQ). The asterisk represents the only protein suggested as a potential biomarker (confirmed using ELISA), PON1. Upregulated proteins are represented by blue columns above the threshold (1.2) and the 26 downregulated proteins are highlighted in orange below the threshold.

PON1 is a glycoprotein enzyme and was recently suggested as a novel potential marker for HCC. This hypothesis was confirmed by using the isobaric tags for relative and absolute quantitation (iTRAQ)-based quantitative proteomics [59]. The level of this protein was decreased significantly in the serum of HCC patients, and the results were confirmed using ELISA and supported by the data portal of TCGA (https://tcga-data.nci.nih.gov/tcga, accessed on 17 October 2022). The authors suggested the presence of 41 differentially expressed proteins during HCC development upon using this quantitative proteomic approach and used the cutoff represented in Figure 6 to describe up- or downregulation. They identified 15 upregulated and 26 downregulated proteins. They selected four potential protein markers: two upregulated proteins, alpha-1-antitrypsin (A1AT) and peroxiredoxin 2 (PRDX2), and two downregulated proteins, PON1 and CRP. The only consistent results of the serum concentration with the iTRAQ were for the PON1 protein.

#### 2.4.4. Growth Factors and Their Receptors

**Figure 7:**
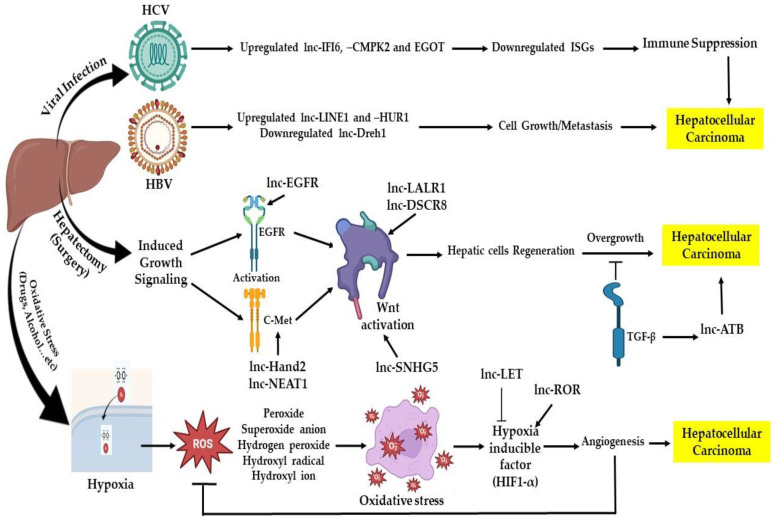
Major growth factor receptor and signaling pathways in HCC. The figure was created using BioRender.com.

In HCC, growth factors and the associated receptors frequently exhibit overexpression or dysregulation, as illustrated in Figure 7 [60].

**A.**  **Transforming growth factor beta (TGFβ)**

TGFβ-1 controls cell proliferation, programmed cell death, differentiation, and growth. Hepatocarcinogenesis is linked to the increased cellular expression of TGFβ-1, which is released by HCC cells. Tumor cells use TGFβ-1 to prevent tumor recognition and promote angiogenesis [61].

**B.**  **Insulin growth factor-1 (IGF-1)**

IGFs I and II are small polypeptides made up of 67 and 70 amino acids, respectively. Due to their strong activity in stimulating growth, both play significant roles in the progression of hepatic carcinogenesis. The emergence of HCC has been linked to the deregulation of IGF-I. Patients with HCC have significantly lower blood concentrations of IGF-I (three times lower) than IGF II compared with the levels in patients without HCC [62,63].

**C.**  **Vascular endothelial growth factor (VEGF)**

This key factor in tumor neovascularization is a homodimer glycoprotein with a molecular mass of 45 kDa. Compared to normal tissues, the expression of VEGF is upregulated in HCC patients’ tissues. The induction of upregulated expression of VEGF and other growth factors by the tumor cells stimulates the formation of new vasculature surrounding the tumor in a process known as angiogenic switch. This phenomenon results in the exponential growth of the tumor, which impairs the prognosis of HCC [64,65].

**D.**  **Hepatocyte growth factor (HGF)**

This factor is expressed and released into the extracellular space by the hepatocyte stellate cells. HGF acts on its receptor, c-Met, on the hepatocytes, promoting several processes including cell division; motility; intracellular invasion; and the development of cancer. When compared to chronic hepatitis as the control group, HGF levels are considerably higher in the HCC group. HGF has been linked to the recurrence of tumors [66].

#### 2.4.5. Cytokines

The growth and regeneration of liver cells, as well as hepatic inflammation, particularly those caused by viral illnesses, fibrosis, and cirrhosis, are pathological processes in which cytokines play a significant role. These small (5–20 kDa) secreted proteins, such as interleukin-6 and interleukin-8, whose high levels have been detected in HCC patients and are linked with the growth of tumors, regulate the immune system and have direct antiviral effects [67]. IL-8 has multiple functions including triggering an immune response in neutrophils, causing enzyme release and the development of surface adhesion molecules. It also directly affects tumor growth by stimulating the proliferation of vascular endothelial cells and facilitating angiogenesis [68,69]. In hepatic cells, IL-8 increases the probability of metastasis and the development of new tumors. Comparing healthy individuals to those with hepatocellular carcinoma, higher levels of IL-8 were observed, which were positively associated with tumor size, portal vein thrombosis, and advanced-stage disease with lymph node metastases. As a result, it might be a possible marker that helps in the diagnosis and prognosis of HCC [70].

#### 2.4.6. Metabolites

Small metabolites with molecular masses less than 2 kDa can be identified, quantified, and characterized accurately using metabolomics of the “omics” approach. Numerous cellular metabolic alterations are caused by HCC that arises from a cirrhotic liver. These alterations produce detectable metabolites in the serum, such as bile, phospholipids, peptides, sphingolipids, reactive oxygen species, amino acids, long-chain carnitines, modified nucleosides like 1-methyladenosine (M1A), and others. Hepatocellular metabolic alterations play a crucial role in cancer progression. For instance, the process of glycolysis in tumors is shifted to synthesize nucleotides necessary for the pentose–phosphate pathway rather than energy production in the form of ATP. However, understanding the metabolomics of tumors is a promising approach to identify potential biomarkers for the early diagnosis of HCC [71,72,73].

#### 2.4.7. Molecular Markers

HCC patients’ blood contains specific nucleic acids, such as DNA, RNA, and nucleosomes. Tumor emergence and growth have been linked to variations in their levels. Short noncoding RNAs called microRNAs (miRNAs) are produced by the transcription of primary, precursor, and mature miRNAs from DNA. By dynamically adhering to the 3′UTR, and 5′UTR coding sections of mRNAs as well as the promoters of genes, these mature molecules control the stability or translation of post-transcriptional gene expression mechanisms. It is possible to diagnose disorders using these circulating molecules. For instance, the plasma level of AFP mRNA has been used as a diagnostic molecular marker for HCC.

As hepatocellular carcinogenesis progressed, it was shown that miRNA levels increased. This finding suggests that miRNAs are involved in the initiation, proliferation, and progression of cancer. The inability to distinguish between HCC and hepatitis, the insufficient sample size, the insufficient number of miRNAs to screen, and the absence of independent validation are all limiting factors of using miRNAs as markers [74].

##### Circulating RNAs

**A.**  **AFP mRNA**

AFP mRNA is a possible marker only present in hepatic cells and could indicate tumor proliferation. The AFP mRNA expression rate exceeds 100% in the advanced stages of HCC and also would predict tumor recurrence following hepatectomy. The use of this marker in the diagnosis of HCC is still up for debate, probably as a result of the fact that it is also detected in a variety of different cancers and noncancerous liver conditions. When AFP mRNA is combined with other markers, it could, therefore, be utilized for diagnosis and prognosis [75,76].

**B.**  **Gamma-glutamyl transferase mRNA (GGT mRNA)**

The family genes of GGT play a critical role in the initiation and progression of several solid tumors. Patients with chronic hepatitis C, benign liver tumors, or liver cancer can also have GGT mRNA in their blood and liver tissues. A, B, and C are three types of GGT mRNA. While type C is synthesized during pregnancy, type A is formed in the normal liver, noncancerous liver disorders, benign tumors, and secondary liver malignancies. Type B, on the other hand, predominates in HCC. As a result, type B mRNA expression in the liver may be a potential marker of liver cancer [77,78].

**C.**  **Toll like receptor (TLR) mRNAs**

Membrane receptors, termed Toll-like receptors (TLRs), are crucial components of the innate immune system. TLRs are type I membrane glycoproteins that have been structurally defined to contain a cytoplasmic Toll/interleukin-1 receptor (TIR) domain, an extracellular leucine-rich repeat (LRR) domain, and a single transmembrane domain. TLRs serve as sensors for a selected group of molecular patterns associated with pathogens as well as for other molecular patterns. In humans, there are 10 identified TLRs and these are heterogeneously localized from the cell membrane (TLRs 1, 2, 4, 5, and 6) to endosomes (TLRs 3, 7, 8, and 9). Guanosine and uridine-containing ssRNAs were both detected by TLR7 at different sites. Both organic and synthetic ligands could activate TLR7, and as a result of research findings, the development of therapeutic strategies that target TLR7 should be improved [79,80].

TLR2 forms heterodimers with either TLR1 or TLR6, depending on the ligand. However, TLRs 3, 4, 5, 7, and 9 are assumed to exert signaling mechanisms through their homodimers after attaching to their respective ligands. In addition to its ligand, TLR4 also needs MD2, a requisite molecule for lipopolysaccharide signaling [81].

##### MicroRNA (miRNA)

Small noncoding RNAs, termed microRNAs (21–23 bases), can reduce or enhance the synthesis of target mRNAs, or they can attach to a specific nucleotide sequence in the 3′-UTR of downstream target mRNAs to increase or decrease protein synthesis. The relationship between miRNA and the progression of tumors is controversial. Several pathways such as apoptosis, differentiation, and cellular proliferation are just a few of the numerous cellular processes that are regulated by the over 500 miRNA genes that have been discovered. miRNA has been linked to both tumor suppression and progression. miRNAs are promising targets for therapeutic applications, prognosis, and as a diagnostic tool [82].

MiR-500 is abundantly expressed in liver cancer cells. It has a strong association with the control of liver growth, which makes it relevant to the emergence of cirrhosis and be a potential biomarker for HCC [83,84]. MiR-21 levels have an 83% specificity and 61% sensitivity for distinguishing HCC from chronic hepatitis. High levels of related miRNA markers such as miR-15b and miR-130b are expressed in HCC. miR-130b shows 88% sensitivity and 81% specificity, while MiR-15b has a 98% sensitivity and a 15% specificity (very low). However, seven miRNAs (21, 223, 26a, 27a, 122, 192, and 801) have been demonstrated to have the strongest diagnostic performances for early-stage HCC associated with HBV [85]. For the early identification of HCC, the measurement of miRNAs (625, 532, 618, 516-5P, and 650) in urine has been employed [86,87].

##### Long Noncoding RNA (lncRNA)

lncRNA are RNA sequences of more than 200 nucleotides exhibiting cellular processes without coding proteins. The biogenesis of these lncRNA is regulated by major events such as epigenetic modification, complexity of transcription, and RNA processing. Epigenetic changes are becoming more prevalent, which is linked to HCC. LncRNA has been discovered to play a significant role in the onset and development of HCC among these alterations due to different abnormal chromatin marks. In tumor tissues, lncRNA levels have either increased or decreased. In the meantime, several lncRNA have been discovered in various bodily fluids. Due to the distinctive expression of lncRNA, the use of lncRNA as a tumor marker is also greater than that of the coding RNA protein. For HCC, lncRNA’s diagnostic performance is quite good. The 2-lncRNA signal has 61% accuracy and 91% specificity for the diagnosis of HCC [88].

##### Circulating Tumor Cells (CTCs)

CTCs are unique blood cells that develop from solid tumors and are thought to be the first signs of metastasis. They have been suggested as a potential alternative strategy for diagnosing and treating HCC metastases. CTCs are released to the circulation; however, they might have two clinical advantages. The first is the correlation between their presence and the target tumor’s stage, size, and metastasis; they offer essential details about the tumor. The second is its potential application as a real-time parameter for monitoring the response to HCC treatment [89].

**Figure 8:**
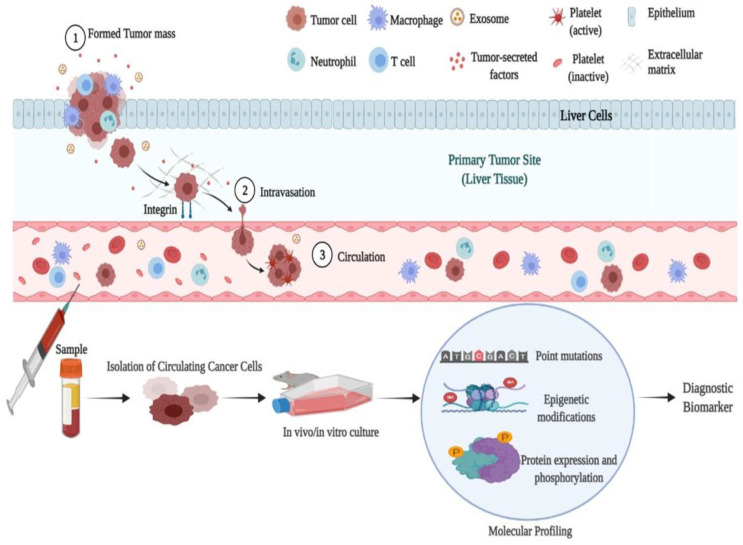
Illustration showing the tumor cell migration to the blood stream and the potential use of these cells in predicting novel biomarkers. The process may start by propagating these cells in vivo/in vitro, followed by studying the molecular profile like mutations, epigenetic, and expression modifications of these cells. The figure was created using BioRender.com.

CTCs can be detected by a simple peripheral blood draw and possibly indicate the comprehensive characteristics of tumor appearances. Numerous platforms using immunoaffinity and biophysical properties to detect CTCs have been established to categorize and capture CTCs with high efficacy. The presence of measurable amounts of CTCs, as well as the biological features and genomic heterogeneousness between the different CTCs, can predict the disease development and HCC treatment responses of patients [90]. Moreover, Zhang et al. [91] demonstrated the usage of CTCs to evaluate the development of hepatocellular carcinoma (HCC). Figure 8 illustrates a potential approach to use CTCs from patients to predict novel biomarker(s) to diagnose HCC.

## 3. Future Perspectives

With the advancement of genomics and proteomics, novel biomarkers will be available for the early diagnosis and surveillance of HCC. Challenges to biomarker studies remain, including incomplete cohort data, selection bias during sample collection, and limited sample size for discovery and validation studies. Given the nature of hepatocellular carcinoma, the complexity of the disease, and the variety of risk factors, it is difficult to identify and use a single and universal biomarker for HCC patients. Recent developments in artificial intelligence and machine and deep learning techniques such as combining biomarkers of multimodal characteristics may play an important role, in the near future, in enhancing the accuracy of prognosis and diagnosis. The success of this new approach will depend on the availability and access of several pieces of information such as human genomic data, analyzed images, and original laboratory and clinical data.

## 4. Conclusions

Several biomarkers are considered to diagnose HCC early, including downregulated or upregulated serum/plasma protein markers secreted during HCC development, circulating nucleic acids or cells, and metabolites. We have summarized the biomarkers in Table A1 and Table A2 and provided a diagnostic model for HCC in Table A3 (available in Appendix A) with aim of providing researchers and clinicians a comprehensive review for further research.

## Data Availability

The data used to support the findings of this study are included within the article.

## Appendix A

**Table A1:** Diagnostic potentials of some markers for diagnosing patients with HCC from liver cirrhosis.

Single Marker	Abb.	Characteristics	AUC	Sn	Sp	Author
Alpha-fetoprotein	AFP	Oncofetal glycoprotein	0.8	59	89	[92]
			0.66	30	100	[27]
			0.76	36	100	[93]
Lens culinaris agglutinin-reactivee AFP	AFP-L3	Glycosylated isoform of AFP	0.67	51	83	[94]
Dickkopf-1	DKK1	Glycoprotein	0.82	68	89	[95]
			0.65	50	81	[96]
Golgi protein 73	GP73	Transmembrane glycoprotein	0.94	92	84	[97]
			0.94	75	97	[98]
Annexin A2	ANXA2	Phospholipid-binding protein	0.8	83	68	[32]
Epithelial membrane antigen	EMA	A mucins transmembrane glycoprotein	0.81	70	78	[35]
Fibronectin	FN	Glycoprotein	0.7	67	82	[35]
Cytokeratin-1	CK1	Cytoplasmic protein	0.83	75	82	[39]
Nuclear matrix protein-52	NMP-52	Nuclear protein	0.72	62	70	[99]
Midkine	MDK	A heparin-binding growth factor	0.81	76	71	[73]
			0.7	71	62	[100]
			0.83	82	84	[101]
Insulin-like growth factor 2	IGF2	Growth factor	0.86	80	73	[102]
Interleukin-6	IL-6	Cytokine	0.78	72	70	[102]
Thioredoxin	TRX	Antioxidant protein	0.79	74	71	[73]
			0.84	75	80	[103]
1-methyladenosine	M1A	Modified nucleoside metabolites	0.75	74	75	[73]
α-1-acid glycoprotein	AGP	Glycoprotein	0.74	74	75	[40]
			0.83			[104]
			0.91	77		[105]
			0.94	71		[106]
C-reactive protein	CRP	Acute-phase protein	0.71	66	75	[40]
			0.76	72	70	[107]
Glypican-3	GPC3	Glycoprotein antigens	0.76	55	84	[92]
			0.79	60	52	[93]
			0.87	68	92	[108]
Osteopontin	OPN	Glycophosphoprotein	0.8	82	65	[109]
			0.99	100	98	[110]
			0.63	46	80	[96]
			0.88	71	80	[111]
Squamous cellular carcinoma antigen	SCCA	Glycoprotein antigens	0.81	89	50	[112]
Des-gamma-carboxy prothrombin or protein induced by vitamin K absence or antagonist II	PIVKA-II (DCP)	Enzymes and isoenzymes	0.71	51	91	[96]
			0.71	71	70	[94]
MicroRNA-21	miR-21	Genetic and molecular marker	0.63	65	63	[113]
MicroRNA-29	miR-29		0.68	62	65	
MicroRNA-200	miR-200		0.72	65	63	
MicroRNA-355	miR-355		0.6	60	55	
MicroRNA-148a	miR-148a		0.92	89	89	[114]
MicroRNA-122	miR-122		0.93	95	81	[115]
MicroRNA-224	miR-224		0.77	85	79	
MicroRNA-301	miR-301		0.84	78	98	[116]

AUC: area under the ROC curve, Sn: sensitivity, Sp: specificity.

**Table A2:** Diagnostic potentials of some combined markers for diagnosing patients with HCC developed from liver cirrhosis.

Combined Marker	AUC	Sn	Sp	Author
AFP + DKK-1	0.76	78	73	[96]
AFP + GPC3	0.85	77	83	[92]
AFP + PIVKA-II	0.76	64	88	[96]
PIVKA-II + DKK-1	0.73	74	73	[96]
AFP + TRX	0.88	82	87	[103]
AFP + OPN	0.74	75	72	[96]
	0.81	86	60	[109]
	0.9	82	77	[111]
Annexin + AFP	0.85	76	81	[32]
AFP + PIVKA-II + OPN	0.73	76	71	[96]
AFP + PIVKA-II + DKK-1	0.75	79	70	[96]
AFP + AGP	0.94	89	90	[105]
C-Myc + P53	0.9	100	87	[117]
miR-200 + AFP	0.7	70	75	[113]
miR-200 + miR-29	0.74	70	65	
miR-200 + miR-29 + AFP	0.84	80	65	
miR-200 + miR-29 + AFP + miR-21	0.88	80	80	
miR-200- miR-29-AFP + miR-21 + miR-355	0.92	90	80	
AFP + miR-122	0.96	98	87	[115]
AFP + miR-224	0.83	94	79	
AFP + miR-301	0.93	90	95	[116]
miRNA-122-5p + miRNA-486-5p + miRNA-142-3p	0.94	80	95	[118]

AUC: area under the ROC curve, Sn: sensitivity, Sp: specificity.

**Table A3:** Diagnostic model for hepatocellular carcinoma diagnosis.

Marker/Model		AUC	Sn	Sp	Author
CK1 score	[1.392 + CK1(μg/mL) × 0.005 + logAFP × 0.141 − albumin (g/L) × 0.032]	0.87	87	77	[39]
FEBA-Test	[0.001 × fibronectin(mg/L) + 0.13 × EMA (μg/mL) + 0.036 × total bilirubin (mg/dL) + 0.83 × logAFP (U/L) − 0.063]	0.92	89	85	[35]
GPC-HCC	[0.517 + 0.018 × GPC3 (ng/mL) + 0.108 × logAFP (U/L) + 0.025 × total bilirubin (mg/dL) − 0.233 × albumin (g/dL)]	0.91	93	91	[93]
HCC-ART	[2.17 + [log(AFP − 1) × 10 × 0.117] + AAR × 0.025 + age × 0.012 + ALP (U/L) × 0.001] − [albumin (g/L) × 0.015]	0.97	96	99	[119]
Simplified HCC-ART	[Age(years) × logAFP (U/L) × AAR × ALP (U/L)]/[Alb (g/L)]	0.92	91	95	[120]
HCC-DETECT	[0.004 × CK-1 (μg/mL) + 0.007 × NMP-52 (μg/mL) + 0.248 × LogAFP (U/L) + 0.951]	0.9	80	92	[99]
3AC	[3.057 + AGP (ng/mL) × 0.199 − albumin (g/L) × 0.052 + LogAFP × 0.136 + CRP (mg/L) × 0.008]	0.95	85	90	[40]
ATM2	[0.533 + 0.146 × logAFP + 0.062 × MDK (ng/mL) + 0.004 × TRX (ng/mL) + 0.009 × M1A (ng/mL)]	0.94	90	88	[73]
AFP- IGF2- IL6- platelet count	1.17 + AFP (U/L) × 0.002 + IGF2 (pg/mL) × 0.001 + IL6 (pg/mL) × 0.008 − platelet count (×10^9^/L) × 0.001	0.95	90	82	[102]
HCC-Mark	[AFP(U/L) × hs-CRP (mg/L)/(Albumin (g/L) × PLT (×10^9^/L))] × 100	0.9	84	80	[107]

AUC: area under the ROC curve, Sn: sensitivity, Sp: specificity. HCC-ART: HCC α-fetoprotein routine test.
